# New York in September

**Published:** 1899-08

**Authors:** 


### NEW YORK IN SEPTEMBER.

NOTHING remains now but the assembling of the delegates and members to the first meeting of our National organization under the broader auspices of its membership extended to all Americas.

Everything points to an unusually large meeting, and there can be nothing in addition desired so far as a broad, generous and comprehensive programme can contribute to its success.

The extreme East promises to be more largely represented than ever before, and among the old familiar faces long active in the parent organization there will be many new ones, whose first opportunity for attendance will be in the metropolis of Greater New York. The middle West will be well represented, unless all signs fail, while the extreme West will have representative delegations to show their interest in its success and advancement. The North and Southwest promise increased attendance, and the gathering of many of the graduates of the older New York schools will bring to this meeting a number who have only held a membership that distance, time, etc., prevented active interest therein. The Middle States and the Lake States, with those central ones east of the Mississippi, should show an outpouring of their members that should outrival in number all previous conventions, and thus fully make up for all absentees, and make sure of a full appreciation of the effort put forth by our New York colleagues for their entertainment.

Secretary Stewart, on whom has fallen the greater burden of the Associations' duties and work, has been active, earnest, and persistently at his post of duty since the closing session at Omaha's splendid meeting. He has been more efficiently aided this year by the resident State secretaries, and these representatives of our National organization should be more carefully selected each year, and their value in the general work broader and better.

A generous welcome is extended to every reputable veterinarian to attend; the doors of membership are wide open to every one possessing the necessary qualifications, and the broader field of duty of our organization will find fitting work for every member to do.

The silver anniversary of the American Veterinary College will be one of many joyful surprises to the alumni of that institution. Every son of the old school should be there to greet their dean and old preceptor. Time makes rapid changes, and the older era of that school, successful in a degree far beyond the usual measure meted out to such an institution, should be closed with all the joyful ceremonies that can be crowded in. Equally important is it to every lover of collegiate advancement that the new step forward in amalgamation of the two oldest schools of our land should be started on its career with all the strength and goodly influences of the alumni of both institutions, that it may make for itself in the field of veterinary science the name and fame of the American Veterinary College of to-day.

